# Acute Brown-Sequard syndrome from an intradural C5–C6 disc herniation: Case report and literature review

**DOI:** 10.1016/j.ijscr.2025.111905

**Published:** 2025-09-03

**Authors:** Youssef Jamaleddine, Majed Ali, Emanuel-Youssef Dib, Chahine Assi, Ramzi Moucharafieh, Mohammad Badra

**Affiliations:** a Department of Orthopedic Surgery, Lebanese American University Medical Center, Beirut, Lebanon; b Department of Orthopedic Surgery, Faculty of Medicine, Balamand University, Beirut, Lebanon; c Department of Orthopedics and Traumatology, Clemenceau Medical Center, Beirut, Lebanon

**Keywords:** Case report, Cervical, Intradural disc herniation, Brown-Sequard syndrome, Anterior cervical discectomy and fusion, Spinal cord compression

## Abstract

**Introduction and importance:**

Brown-Sequard syndrome (BSS) is a rare spinal cord hemisection syndrome characterized by ipsilateral weakness with contralateral loss of pain and temperature. Cervical intradural disc herniation (CIDH), an extremely rare phenomenon, is an uncommon etiology of BSS. Only around 50 cases of CIDH have been reported in the literature to date, including our own.

**Case presentation:**

A previously healthy 45-year-old man presented with sudden right-sided hemiparesis and contralateral sensory loss, consistent with incomplete BSS. MRI revealed a large C5-C6 disc extrusion penetrating the posterior longitudinal ligament and breaching into the intradural space, compressing the right anterolateral spinal cord. Urgent anterior cervical discectomy and fusion (ACDF) was performed, with intradural disc removal and dural repair. Neurological improvement was immediate. At 12 months, the patient had fully recovered motor and sensory function.

**Discussion:**

BSS is the most frequent presentation of CIDH, yet preoperative diagnosis is uncommon (around 13 %). Suggestive MRI features include the Y-sign (bifid ventral dura) and a CSF halo around the fragment, but confirmation is often intraoperative. An anterior approach (ACDF) allows definitive decompression, intradural fragment removal, and dural repair; early surgery is associated with favorable outcomes, as mirrored by this patient's course.

**Conclusion:**

CIDH presenting as BSS is exceedingly rare but should be considered in lateralizing cervical myelopathy. Prompt anterior decompression with dural repair can result in excellent outcomes.

All the information in this manuscript has been reported in accordance with SCARE criteria.

## Highlights

• Cervical intradural disc herniation with Brown-Sequard syndrome is extremely rare.
• Anterior C5-C6 discectomy with dural repair led to rapid neurologic recovery.
• MRI Y-sign and CSF halo aided pre-operative diagnosis of intradural fragment.
• Literature review finds fewer than 50 cervical intradural disc herniation cases.
• Early surgery for Brown-Sequard syndrome enables full recovery within 12 months.

## 1 Introduction

Intradural disc herniations (IDH) are exceptionally rare with the vast majority occurring in the lumbar spine (>90 %), while cervical IDHs (CIDH) represent approximately 3 % of all IDH cases [1]. This finding is also supported by a literature review which contextualizes CIDH as a notably rare entity [2]. CIDH was first reported in 1959, and to date, approximately 50 cases have been documented in the literature [3].

While classically associated with trauma, Brown-Sequard syndrome (BSS) has also been linked to tumors, ischemia, and, rarely, disc herniation [4]. Among 23 CIDH cases studied in a systematic review, BSS (including complete BSS, incomplete BSS, and BSS combined with Horner's syndrome or radiculopathy) was the most common clinical presentation, found in over half of reported cases [1].

The pathophysiology of CIDH often involves adhesions between the posterior longitudinal ligament (PLL) and dura, sometimes in the setting of ossification of the PLL (OPLL), which may predispose to dural penetration by an extruded disc [5,6]. However, spontaneous CIDH without trauma or OPLL has also been documented [7]. Preoperative diagnosis is often challenging, with only 13 % of CIDH cases correctly identified on imaging prior to surgery in a systematic review done on 23 cases of CIDH [1]. MRI findings such as the “Y-sign,” “halo sign,” and “hawk-beak sign” may aid in diagnosis but are not pathognomonic [1].

Surgical management is urgent. Anterior cervical approaches allow access to the disc and dura, with most cases treated via ACDF or corpectomy and dural repair [1,2]. Favorable neurological outcomes have been reported when decompression is performed promptly [1].

In this article, we present a case of non-traumatic C5-C6 intradural disc herniation manifesting as spontaneous incomplete Brown-Sequard syndrome, along with a supporting review of the literature.

## 2 Case presentation

### 2.1 Patient information

A 45-year-old previously healthy right-handed man presented to the emergency department with sudden-onset, debilitating right shoulder and posterior scapular pain. He noted profound weakness in the right upper and lower extremities accompanied by numbness and loss of pain and temperature sensation on the contralateral (left) side of his body. He denied antecedent trauma, constitutional symptoms, or bowel and bladder dysfunction.

### 2.2 Clinical findings

Neurological examination revealed flaccid paresis of 2/5 in the right upper and lower extremities, whereas left-sided motor strength was preserved at 5/5. Sensory testing showed diminished pain and temperature sensation on the left side, with preserved proprioception and vibration bilaterally. Hyper-reflexia and an extensor plantar response were present on the right; reflexes on the left were normal. The constellation of signs was consistent with an incomplete Brown-Sequard syndrome.

### 2.3 Diagnostic assessment

**Fig. 1:**
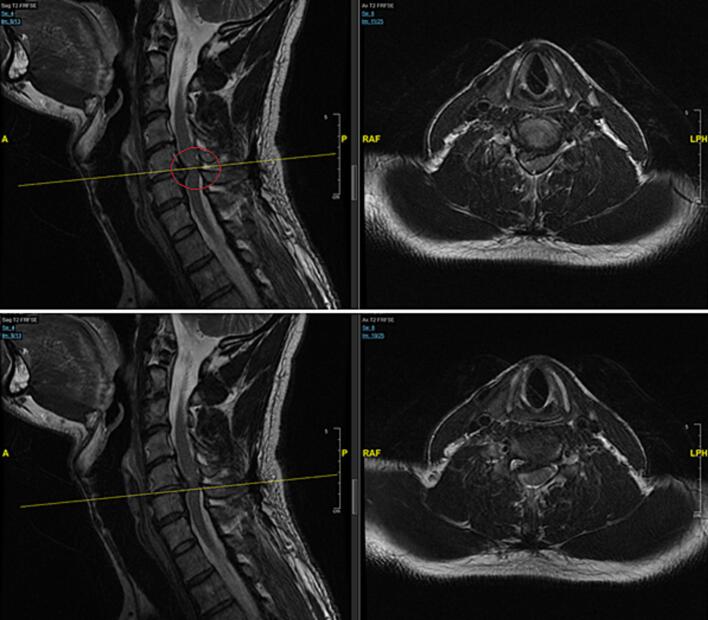
Sagittal and axial T2-weighted MRI images of the cervical spine (3T) demonstrating a large, right paracentral disc extrusion at the C5–C6 level. The disc fragment breaches the posterior longitudinal ligament and exerts significant mass effect on the right anterolateral aspect of the spinal cord, suggestive of intradural extension. The red circle highlights the halo sign. (For interpretation of the references to colour in this figure legend, the reader is referred to the web version of this article.)

Urgent brain MRI yielded no acute ischemic or hemorrhagic pathology, effectively ruling out cerebrovascular etiologies. Cervical spine MRI demonstrated a substantial right paracentral extruded disc fragment at C5-C6 breaching the posterior longitudinal ligament (PLL) and protruding into the intradural space (Fig. 1). The fragment produced severe mass effect on the right anterolateral aspect of the spinal cord without overt myelomalacia. Small, clinically insignificant disc bulges were also noted at C3-C4 and C4-C5. No facet-joint disease or spinal instability was identified. Collectively, these findings are diagnostic of an intradural C5-C6 disc herniation.

### 2.4 Therapeutic intervention

**Fig. 2:**
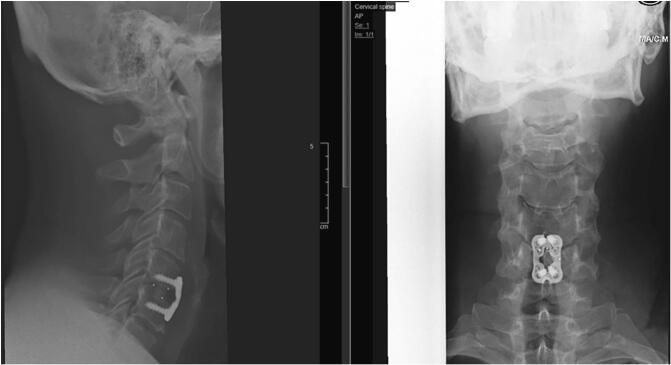
Postoperative anteroposterior and lateral radiographs of the cervical spine demonstrating satisfactory placement of the interbody cage and anterior cervical plate at the C5–C6 level, with appropriate alignment and no evidence of hardware complication.

Given the acute and progressive neurological deficits, the patient was transferred urgently to the operating room for an anterior cervical discectomy and fusion (ACDF) at C5-C6 via a left-sided Smith-Robinson approach. After standard exposure, the intervertebral disc was incised and evacuated with pituitary rongeurs and curettes. The PLL was opened on the left, exposing the dura. While decompressing toward the right, a free fragment was visualized within the intradural space and removed. A brisk cerebrospinal fluid (CSF) egress ensued, revealing a ventral dural defect. Meticulous inspection with a blunt nerve hook confirmed complete spinal cord decompression. The dural breach was repaired with a dural patch (DuraGen®) supplemented by fibrin glue dural sealant. A cage packed with allograft was inserted, and an anterior cervical plate with screws was applied for stabilization (Fig. 2). No drain was placed, and layered closure was performed.

### 2.5 Follow-up and outcomes

**Fig. 3:**
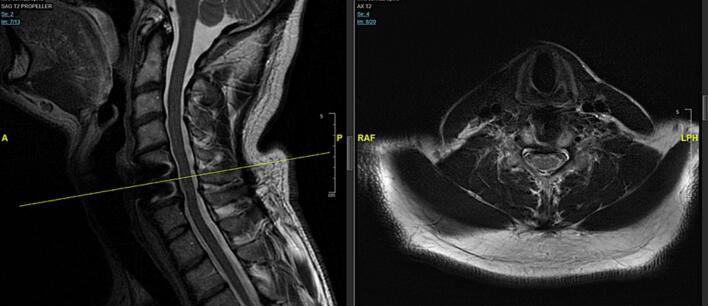
Follow-up sagittal T2-weighted MRI of the cervical spine obtained 3 years and 6 months postoperatively, demonstrating solid fusion at the C5–C6 level with no recurrent disc herniation or spinal cord compression.

Postoperatively, the patient exhibited immediate improvement in right-sided strength. By postoperative day 2 he ambulated with assistance, and there was no evidence of CSF leakage. At the two-month follow-up he had regained full motor strength (5/5) in all extremities. Residual paresthesias were managed effectively with pregabalin 75 mg twice daily. At one year, neurological examination was normal apart from mild, tolerable persistent numbness. Cervical radiographs confirmed solid fusion without hardware complications. Follow up MRI of cervical spine was repeated 3 years 6 months post-op, showing no recurrent disc herniation or significant spinal cord compression (Fig. 3).

## 3 Discussion

Our patient's presentation with acute incomplete BSS was consistent with the most common clinical manifestation of CIDH [1,8]. The clinical presentation of cervical intradural disc herniation varies, but the most commonly reported syndrome is Brown-Sequard syndrome (BSS), either in complete or incomplete form. This reflects the typical unilateral compression of the spinal cord by an intradural fragment, producing ipsilateral motor weakness and contralateral loss of pain and temperature sensation. In the systematic review by Guan et al., BSS was observed in over half of reported cases [1]. Other clinical presentations include quadriplegia, gait or finger ataxia, radiculopathy, and nuchal pain, with Brown-Sequard syndrome occurring in about 43.2 % of cases and Horner's syndrome in about 10.8 % [1]. In one illustrative case, a patient initially developed neck pain and tingling before progressing over a day to bilateral quadriparesis and voiding difficulty, highlighting how early radicular symptoms can evolve rapidly toward severe myelopathy [10]. Such variability in presentation often leads to diagnostic delay or initial misdiagnosis as more common spinal pathologies, including tumors or demyelinating disease. Being aware of this variability, particularly in patients presenting with acute, asymmetrical myelopathy or hemicord signs without trauma, is essential.

The level of our patient's herniation was consistent with the literature, as supported by a 2024 review, which identified C5-C6 as the most common site of cervical IDH (44.9 %), followed by C6-C7 (20.4 %), C4-C5 (18.4 %), C3-C4 (12.2 %), and C7-T1 (4.1 %) [3].

Although the exact pathophysiology of CIDH remains unclear, proposed mechanisms include adhesions between the posterior longitudinal ligament and the dura mater, ossification or hypertrophy of the PLL, congenital anatomical anomalies, and trauma. These factors may predispose the dura to weakening or rupture, facilitating intradural migration of disc material [9,10].

Diagnosing CIDH preoperatively remains a significant challenge due to its rarity and overlap with more common spinal pathologies. Cervical disc herniation classically presents as radiculopathy or myelopathy, similarly to other diagnoses, including extramedullary spinal tumors (e.g., meningiomas, schwannomas), epidural hematoma, transverse myelitis, and cervical spondylotic myelopathy [3]. CIDH, on the other hand, can present with more severe neurological signs and symptoms like Brown-Sequard syndrome or Horner's Syndrome [3]. Therefore, it is critical to understand the differential diagnosis when dealing with such cases.

MRI remains the primary imaging modality for initial evaluation; however, conventional findings may be nonspecific. A literature review published in 2019 identified four uncommon imaging signs that may raise suspicion for cervical IDH, which include the hawk-beak sign, halo sign, Y-sign, and epidural gas sign [2]. In our case, the intradural location of the herniated fragment was suggested preoperatively by the presence of a bifid ventral dural line on sagittal T2 MRI (Y-sign), as well as a rim of cerebrospinal fluid around the disc fragment on axial images (halo sign). Additionally, another review article emphasized on the halo and Y-signs as key indicators of cervical IDH [10]. These signs are increasingly recognized but remain subtle, contributing to the low rate of preoperative diagnosis [1].

Ultimately, diagnosis is most often confirmed intraoperatively, as imaging signs can be subtle or absent. In a systematic review done on 23 cases of CIDH, only 13 % of cervical intradural disc herniation cases were diagnosed preoperatively, while 87 % were confirmed during surgery [1]. Direct visualization of disc material breaching the dura establishes the diagnosis and may retrospectively validate subtle MRI findings. This highlights the importance of maintaining suspicion when clinical and radiologic features are discordant [1].

In our case, anterior decompression allowed successful removal of the disc fragment and visualization of the dural tear. Prior studies have demonstrated that anterior cervical discectomy and fusion is the more preferred surgical approach and offers better surgical access and outcomes compared to posterior decompression [2]. By providing direct access to the affected disc, the anterior approach enables more effective removal of herniated material and facilitates dural repair [10]. Cases in which the posterior approach was preferred, were justified by the IDHs transmigration to the back causing compression to the dorsal cord [7]. The posterior approach, in which a dural incision is made to remove the hernia, carries the risk of spinal cord injury [1]. Most of the cervical IDHs in the literature were treated surgically via an anterior approach, but a larger portion of patients who underwent a posterior approach had improved recovery [8]. One potential complication, regardless of either anterior or posterior approach, is the possibility of persistent CSF leak and eventual fistula [8]. The most preferred surgical approach for CIDH remains the anterior discectomy and fusion [3].

The use of a dural substitute and fibrin sealant (DuraGen® patch and fibrin glue in our case), was consistent with techniques described in other CIDH reports [8]. We achieved watertight closure with no postoperative CSF leak.

The patient's rapid and complete neurological recovery was consistent with published outcomes. In one systematic review, all but one of the 23 CIDH patients had postoperative neurological improvement, and nearly half achieved complete recovery (43 %) [1]. Similarly, another review done on 37 cases of CIDH emphasized that early surgical decompression is associated with highly favorable neurological outcomes, particularly in patients presenting with incomplete syndromes like BSS [8]. They also found that full recovery was not associated with patient age, as patients at both ends of the age range attained complete recovery [8]. Collectively, these findings support the observation in our patient and underscore the importance of early diagnosis and expeditious management in optimizing outcomes for CIDH-associated BSS.

While thoracic IDH has been reported slightly more frequently than cervical cases, cervical intradural disc herniation (CIDH) remains significantly rarer and less well-characterized. CIDH accounts for approximately 3 % of all intradural disc herniations, compared to 5 % in the thoracic spine and 92 % in the lumbar region [11]. This rarity, combined with the more compact anatomy of the cervical spinal canal, may increase the risk of rapid neurological deterioration and underscores the importance of early detection and surgical intervention.

Clinical presentation varies significantly by spinal level. Thoracic IDH typically causes spastic paraparesis, whereas CIDH more often presents with Brown-Sequard syndrome due to hemicord compression [12]. In contrast, lumbar intradural disc herniations usually result in radiculopathy or cauda equina syndrome, reflecting involvement of nerve roots rather than the spinal cord. Those with cervical or thoracic lesions frequently exhibit profound myelopathy, given the direct compression of the cord, whereas lumbar lesions tend to spare the cord and involve lower motor neuron pathways [11,12].

The rarity of thoracic IDH is attributed to the low incidence of adhesions between the ventral dura and the posterior longitudinal ligament in the thoracic region, which reduces the likelihood of disc material penetrating the intradural space [12].

In conclusion, CIDH is a rare but important differential diagnosis in patients with acute BSS. MRI signs may aid in diagnosis, but definitive identification is often intraoperative. Anterior cervical decompression with fragment removal and dural repair is an excellent approach and is associated with excellent outcomes.

### 3.1 Limitations

This is a single case report and, as such, its findings are not generalizable and larger case series are needed.

This case report has been reported in line with the most recent SCARE checklist [13].

## Author contribution

Youssef Jamaleddine, Majed Ali, Emmanuel-Youssef Dib: Writing and drafting the article.

Chahine Assi, Ramzi Moucharafieh, Mohammad Badra: Critical revision.

All authors provided final approval of the version to be submitted.

## Consent

The patient in this manuscript has given written informed consent to publication of their case details.

## Ethical approval

Since we reported the patient anonymously and since we took approval of the concerned patient, this case is exempted from IRB approval at CMC.

## Guarantor

Youssef Jamaleddine, Mohammad Badra.

## Research registration number

N/A.

## Funding

None.

## Conflict of interest statement

None.
